# Ovulatory and anovulatory cycle phase influences on QT interval dynamics during the menstrual cycle

**DOI:** 10.1371/journal.pone.0320846

**Published:** 2025-05-16

**Authors:** Borna Naderi, Lauren Yee, Sonia Shirin, Jerilynn C. Prior, Christopher Cheung, Brianna Davies, Andrew D. Krahn

**Affiliations:** 1 Heart Rhythm Services, Division of Cardiology, Department of Medicine, University of British Columbia, Vancouver, British Columbia, Canada; 2 Centre for Menstrual Cycle and Ovulation Research, Division of Endocrinology, Department of Medicine, University of British Columbia, Vancouver, British Columbia, Canada; 3 Women’s Health Research Initiative, Vancouver, British Columbia, Canada; 4 School of Population and Public Health, University of British Columbia, Vancouver, British Columbia, Canada; UPR: University of the Poonch Rawalakot, PAKISTAN

## Abstract

**Background:**

Ovarian hormones affect cardiovascular health yet few sufficient-sized studies with reliable ovulatory documentation have assessed the QTc-hormonal relationship. This study investigated QTc changes across ovulatory and anovulatory menstrual cycle phases.

**Methods:**

This prospective cohort investigation, a cardiac sub-study of the Menstruation and Ovulation Study 2 (MOS2), involved 62 healthy, regularly menstruating community-dwelling women during spontaneous menstrual cycles. Electrocardiographic recordings were obtained within-woman during different cycle phases: mid-follicular for all, and luteal (ovulatory) or premenstrual (anovulatory), documented by the validated Quantitative Basal Temperature^©^ method. Fridericia’s formula rate-corrected the QT interval (QTc). A subsequent meta-analysis was conducted, pooling data from three additional studies to evaluate ovulatory follicular-luteal phase QTc changes.

**Results:**

In the 26 ovulatory cycles, QTc minimally decreased from the mid-follicular to the luteal phases (383.0 ± 12.8 vs 382.6 ± 12.8 msec, *P* = .859). QTc in the 36 anovulatory cycles tended to increase from mid-follicular to premenstrual phases (381.7 ± 13.1 vs 385.0 ± 16.1 msec, *P* = .166). The meta-analysis in ovulatory cycles yielded a random-effects weighted mean QTc shortening of 1.67 msec (*P* = .53) in the luteal vs the follicular phase, aligning with our cohort data.

**Conclusion:**

In confirmed ovulatory cycles, QTc changes were minimal, showing no meaningful luteal phase QTc shortening. QTc changes in anovulatory cycles were also insignificant, with a small QTc prolongation likely due to longer estradiol exposure not counterbalanced by progesterone. Under normal physiological conditions, QTc changes during the menstrual cycle are trivial, and menstrual status does not need to be considered when interpreting the QT interval.

## Introduction

Ovarian hormones play a major role in cardiovascular health [1]. These hormones fluctuate during the menstrual cycle and bring about reasonably predictable physiological changes for women or people with ovaries of reproductive age (PORA). Reproductive life (duration of menstruation) spans 25–45 years, with an average of 36 years from menarche to menopause that occurs on average at 51 years of age [2,3]. Menstruation is variable in length, ranging between 21 and 35 days between and within women. However, regular and month-apart menstrual cycles may be ovulatory (releasing an egg and producing high progesterone levels) or anovulatory [4]. If a cycle is ovulatory, it can be separated into two hormonally different phases: follicular phase (FP) that has increasing estradiol levels and the luteal phase that has high progesterone and moderate estradiol (LP).

The corrected QT interval (QTc) is an important cardiovascular measure that is used for ventricular arrhythmia risk stratification [5]. QTc is subject to a complex array of influences throughout the menstrual cycle, including hormonal fluctuations and autonomic nervous system modulations. Techniques like double autonomic blockade [6,7], and pharmacological studies involving exogenous hormones can remove that [8,9], thereby accentuating the hormonal impact on the QTc. However, it is essential to also study these changes under natural menstrual physiological conditions. Thereby, the association between menstrual cycle phases and QTc warrants investigation [6,10,11].

Most menstrual cycle QTc studies are small (*i.e.,* < 15 women) [10], and few reliably document ovulation using cycle-timed serum progesterone level (ovulatory threshold of ≥9.5 nmol/L; ≥ 3 ng/ml) [12] or through Quantitative Basal Temperature© (QBT©) changes; a less accurate method is serial urines for LH surge [13]. Regular, normal-length cycles may be normally ovulatory (by QBT© with ≥10 day LP; short LP < 10 days), or anovulatory with silent ovulatory disturbances [4,12,14], most often related to common everyday stressors [15]. The hormonal relationship of QTc changes during cycles of similar lengths that are documented to be of both ovulatory and anovulatory types allows inferences about the effects of low progesterone levels in the premenstrual phase of anovulatory cycles, since estradiol levels remain very similar in the two cycle types [12,16].

Therefore, this study was designed prospectively, with the hypothesis that the follicular phase will have a longer QTc interval than will the luteal phase, based on limited previous literature. The primary objectives were to: 1) compare QTc within-woman between the mid-FP and LP in ovulatory cycles by validated QBT© method [17,18]; 2) assess QTc difference between the FP and premenstrual phases within-woman during anovulatory cycles; and 3) perform a meta-analysis, combining our findings with the prior QTc literature in confirmed ovulatory cycles.

## Methods

### Study population

This prospective cohort study recruited community-dwelling women from the primary “Menstrual Cycle and Ovulation Study 2” (MOS2) for inclusion in this prospective cardiac sub-study [19]. Participants were eligible if they were: 1) women or born with ovaries; 2) age 19–35 years; and 3) menstruating in the last three months with about one month apart cycle lengths. We excluded those using exogenous hormones, including combined hormonal contraceptives in the past three months.

Recruitment occurred between February 3^rd^, 2020, and September 15^th^, 2021, with informed written consent obtained from all participants. Ethics approval was granted by the UBC Clinical Research Ethics Board (*H19 02983*).

### Data collection

Data for this prospective observational trial were gathered over a period of 30–38 days, with a total of two ECGs, one for each cycle phase, recorded per participant. The first ECG recording coincided with the FP. The second ECG recording was timed for after the participant reported a sustained rise in first-morning temperatures (LP) or, lacking that, in the 3^rd^ week of the cycle. Collection was avoided three days before expected flow. The ECGs were recorded in a warm environment by a trained researcher in a previously rested participant.

### Quantitative basal temperature^©^

Through the QBT© assessment, monitoring first-morning awakening temperatures confirmed the presence/absence of ovulation and measured LP length, which has been validated in a blinded comparison with the serial *serum* LH peak [17]. This approach relies on the effect of high luteal phase progesterone to cause a hypothalamic increase in steady-state first morning temperature. All participants’ temperatures were measured using the same batch digital thermometer with a validated precision of ± 0.1°C.

### Diary and questionnaire

Participants menstrual cycle experiences were recorded using the Menstrual Cycle Diary© (S1 Appendix), which offered a structured way to document cycle lengths and a place to record daily first morning temperatures. Other Diary variables were not included in this sub-study. An ECG Questionnaire was also administered prior to each ECG recording to account for variables such as caffeine intake, smoking status, medication use, and recent physical exertion (S2 Appendix).

### Electrocardiographic recordings

ECGs were recorded using the 6-lead AliveCor® KardiaMobile device, a validated alternative to the traditional 12-lead standard ECG [20,21]. Using the Hearts in Rhythm Organization (HiRO) ECG reading tool [22], QT and RR intervals were measured by two readers that were blinded to cycle phase. Two consecutive beats were measured and averaged, with any discrepancies in tracing resolved through consensus. QT intervals were primarily read in lead II using the maximal slope to direct the tangent technique to effects of longer estradiol exposure not counterbalanced by progesterone [23,24]. In situations where the tracing was not of sufficient amplitude or clarity for accurate measurement (<10%), an alternative high-amplitude lead, free of artifact, was selected (typically lead V_5_ or V_6_) [5]. To ensure consistency, the same lead was used for subsequent within-participant tracings.

### Corrected QT interval

Fridericia’s formula (QTc = QT/RR^1/3^) was used to correct the QT interval because of its accuracy across varying heart rates, reduced variability, and the recent recommendations as a more suitable method for clinical QTc investigations [25–27].

### Statistical analysis

Independent Sample T-test, Paired Two-sided T-Test, Mann-Whitney U Test were applied to assess continuous variables, and categorical variables were compared with the Chi-squared test. In addition to the primary analysis, a multiple linear regression analysis (S3 Appendix), QTc directionality change analysis (S4 Appendix), and sensitivity analysis (S5 Appendix) were also conducted. Statistical analyses were performed using SPSS version 29 (Armonk, NY: IBM Corp) and R version 4.3.0.

### Meta-Analysis

Eligible studies were selected based on the following criteria: 1) documented ovulation by a valid method (cycle-timed measures of progesterone ≥ the ovulatory threshold (3 ng/ml or 9.5 nmol/L), presence of urine LH surge, or a quantitative evaluation of a progesterone bioeffect such as QBT©); 2) documented QTc during FP and LP of the menstrual cycle in women with no history of cardiac diseases or health issues; 3) used Fridericia rate correction or data which could be re-calculated to this rate-correction metric; and 4) involved no use of medications known to shorten or prolong the QT interval, or hormonal contraceptives. All continuous variables were reported as mean values with either standard deviations (SDs) or standard errors of the mean (SEMs) which were converted to SD [28].

When primary authors did not provide the necessary QT and RR intervals, the values were deducted from published graphs using ImageJ [29]. This technique involved measuring the pixels corresponding to the lengths of the graph’s columns or error bars and correlating these measurements with the Y-axis scale to estimate the QT/RR interval. The pooled estimate of absolute change (mean and 95% confidence interval [CI]) for FP and LP QTc was computed using the Cochrane analysis tool Review Manager (RevMan 5.4; Computer program) with a random-effects model. Statistical heterogeneity was assessed using the I-squared (I^2^) statistic. In the two-sided analyses, a *P* value of less than.05 was considered statistically significant.

## Results

### Baseline population characteristics

**Fig 1:**
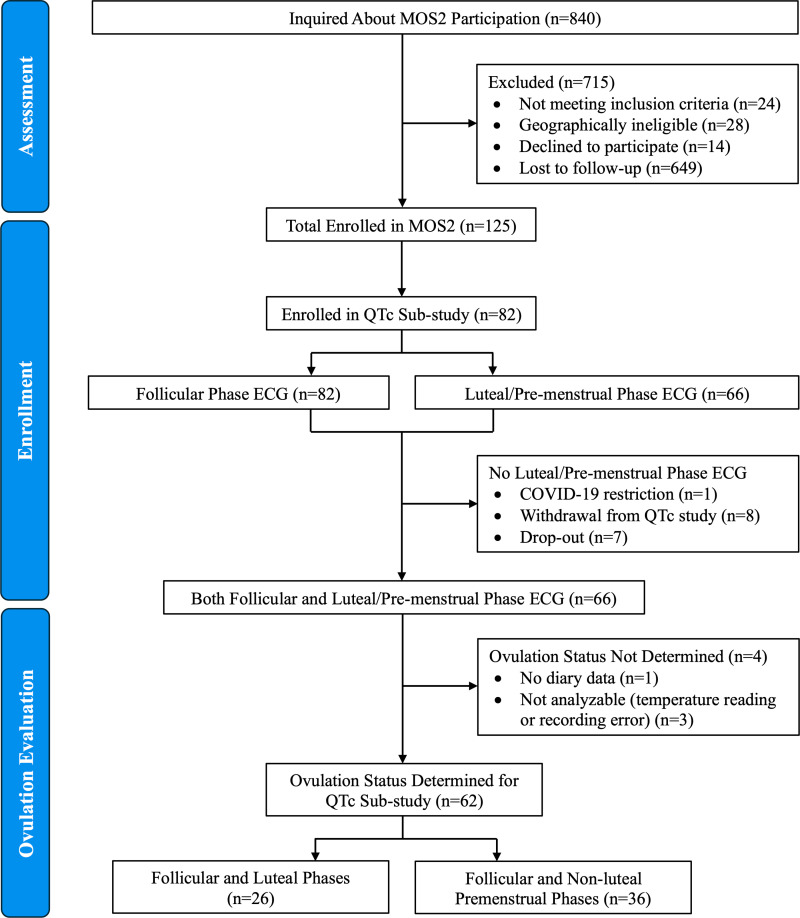
Consort-like Figure Showing the Flow of Participants from the Menstruation and Ovulation Study 2 who volunteered for the QTc Sub-study and those with eligible Paired Follicular and Luteal or Anovulatory Premenstrual information.

A total of 82 women were enrolled from MOS2 in this cardiac sub-study (Fig 1). Of the 62 with complete paired data, 26 women were ovulatory (17 normal LP lengths ≥10 days; nine short LP lengths of <10 days); 36 were anovulatory. Participants in this MOS2 sub-study did not differ from the full cohort except in having slightly higher BMI values (Table 1). The high proportion of anovulatory women was attributed to multiple stressors related to the concurrent SARS-COV-2 pandemic.

**Table 1:** Comparison of Demographic, Anthropomorphic, & Reproductive Variables between Cardiac Sub-study & MOS2 only Participants during SARS-CoV-2 Pandemic.

Parameter		QTc Sub-study N = 62			MOS2 N = 46			Statistical Test	
		N (%)	Mean ± SD	95% CI	N (%)	Mean ± SD	95% CI	P Con†	P Cat^
Age (years)			28.8 ± 4.1	27.8; 29.9		28.5 ± 3.8	27.4; 29.6	.727	
Height (cm)			162.4 ± 7.6	160.5; 164.3		164.8 ± 7.6	162.5; 167.0	.147	
Weight (kg)			64.9 ± 12.4	61.8; 68.1		62.7 ± 12.0	59.1; 66.2	.381	
Body Mass Index (kg/m2)			24.6 ± 4.1	23.5; 25.6		23.1 ± 4.0	21.9; 24.3	**.050**	
Waist Circumference (cm)			78.4 ± 10.9	75.7; 81.2		76.1 ± 10.3	73.0; 79.1	.345	
Ethnicity	White	35 (56.5)			25 (54.3)				.248
	East Asian (e.g., Chinese, Korean)	9 (14.5)			12 (26.1)				
	Other or mixed	18 (29.0)			9 (19.6)				
Living Situation	Alone	12 (19.4)			6 (13.0)				.384
	With ≥1 adult	50 (80.6)			40 (87.0)				
Years of Education			15.6 ± 0.9	15.4; 15.9		15.5 ± 1.1	15.1; 15.8	.425	
Occupation	Full time	32 (51.6)			17 (37.0)				.147
	Student (±part-time)	14 (22.6)			9 (19.6)				
	Part-time and other	16 (25.8)			20 (43.5)				
Regular exercise participation (yes)		40 (64.5)			25 (54.3)				.286
Menarche Age (years)			12.4 ± 1.4	12.1; 12.8		12.5 ± 1.9	11.9; 13.0	.847	
Parity	Nulliparous	56 (90.3)			43 (93.5)				.730
	Parous	6 (9.7)			3 (6.5)				
Cycle Length (days)			29.5 ± 3.7	28.5; 30.4		29.7 ± 3.9	28.6; 30.9	.958	
% Normally ovulatory		24 (38.7)			16 (34.8)				.676

†denotes a Mann-Witney U Test and ^ denotes a Chi-Square Test.

Table 2 outlines the characteristics of the 62 participants with an average age that was similar across ovulatory and anovulatory groups (29.0 years, IQR 21–35). The anovulatory group tended to a higher mean BMI, weight, and waist circumferences and greater education. Parity (childbirth) was greater in those with anovulatory cycles (*P* = .035).

**Table 2:** Characteristics of Participants in this QTc Sub-Study of Menstruation and Ovulation Study 2 (MOS2) during the SARS-COV-2 Pandemic in all with Paired Follicular-Luteal Data (ovulatory), and those with Paired Follicular-Premenstrual (anovulatory) Cycle Data.

		All in the QTc Study n = 62	Ovulatory n = 26	Anovulatory n = 36	*P*-value
**Age (years)**		29.0 (21 - 35)	29.0 (21 - 35)	29.0 (22 - 35)	.983**^**
**Height (cm)**		162.4 (160.5; 164.3)	161.2 (158.2; 164.3)	163.2 (160.7; 165.8)	.304*
**Weight (kg)**		63.5 (44.9–102.0)	57.8 (46.0–78.2)	66.3 (44.9–102.0)	.064**^**
**BMI**		24.6 (23.5; 25.6)	23.7 (22.0; 25.4)	25.2 (23.9; 26.6)	.145*
**Waist Circumference (cm)**		75.5 (61.0–124.2)	74.8 (61.0–91.5)	78.8 (67.0–124.2)	.089**^**
**Ethnicity**	White	35 (56.5)	17 (65.4)	18 (50.0)	.515**†**
	East Asian: Chinese, Japanese, Korean	9 (14.5)	3 (11.5)	6 (16.7)	
	Others or Mixed	18 (29.0)	6 (23.1)	12 (33.3)	
**Living Situation**	Alone	12 (19.4)	5 (19.2)	7 (19.4)	.983**†**
	With ≥1 adult	50 (80.6)	21 (80.8)	29 (80.6)	
**Occupation**	Full-Time	32 (51.6)	14 (53.8)	18 (50.0)	.889**†**
	Student (±part-time)	14 (22.6)	5 (19.2)	9 (25.0)	
	Part-time and Other	16 (25.8)	7 (26.9)	9 (25.0)	
**Years of Education**		16 (12 - 16)	16 (12 - 16)	16 (13 - 16)	**.039^**
**Regular exercise participation (yes)**		40 (64.5)	18 (69.2)	22 (61.1)	.510**†**
**Cycle Length (days)**		29.5 (28.5; 30.4)	29.6 (28.1; 31.0)	29.4 (28.1; 30.7)	.869*
**Luteal Phase Length in Ovulatory Cycles (days)**		--	10.6 (9.7; 11.5)	--	--
**Menarche age (years)**		12.0 (8.0–16.0)	13.0 (9.0–15.0)	12.0 (8.0–16.0)	.083**^**
**Parity**	Nulliparous	56 (90.3)	26 (100)	30 (83.3)	**.035†**
	Parous	6 (9.7)	0 (0)	6 (16.7)	

*Independent Sample T-test and Mean (95% CI), ^Mann-Whitney U Test and Median (min - max), †Chi-Square Tests and n (%) where appropriate.

### ECG/QTc comparisons

**Fig 2:**
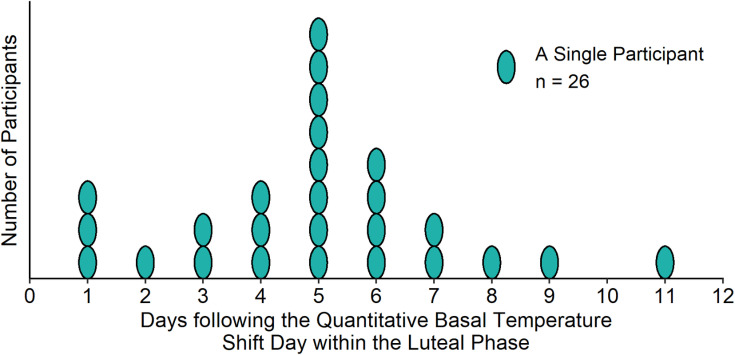
Timing of QTc Measurements in the Luteal Phase of Ovulatory Cycles. Distribution of QTc Measurement Timing Within the Luteal Phase in Ovulatory Cycles by Quantitative Basal Temperature© Analysis in the MOS2 QTc Sub-Study.

Fig 2 illustrates the QTc timing within tested LP’s. Tables 3 and 4 summarize ECG parameters and QTc comparisons respectively. Overall, there were minimal changes in the QTc interval within ovulatory comparisons, and between ovulatory and anovulatory study subjects. The QTc in ovulatory cycles was similar from FP to LP (383.0 vs 382.6 msec, *P* = .859). Conversely, the QTc from the FP to the premenstrual phase was numerically longer in anovulatory cycles (381.7 vs. 385.0 msec, *P* = .166). Across all 62 participants, and both cycle types, the average QTc difference from FP to LP/premenstrual phase was not different (3.68 msec, 95% CI -2.9;10.3, *P* = .268).

**Table 3:** Follicular & Luteal (or Premenstrual) Phase Electrocardiographic Parameters and QTc of Women by Menstrual Cycle Phase within Ovulation Categories in QTc Sub-study of the MOS2 during the SARS-COV-2 Pandemic.

		Complete Cohort	Ovulatory	Anovulatory	*P *value*
**Follicular Phase**	*Population*	n = 62	n = 26	n = 36	–
	QT interval (msec)	362.4 (356.5; 368.2)	369.4 (361.0; 377.8)	357.3 (349.3; 365.2)	**0.038**
	HR (bpm)	72.7 (70.2; 75.2)	69.0 (65.9; 72.0)	75.4 (71.9; 79.0)	**0.010**
	QTc (msec)	382.2 (379.0; 385.5)	383.0 (377.8; 388.1)	381.7 (377.3; 386.1)	0.704
**Luteal/Pre-menstrual Phase**	*Population*	n = 62	n = 26	n = 36	–
	QT interval (msec)	353.7 (347.5; 359.8)	358.7 (350.7; 366.8)	350.0 (341.0; 359.0)	0.163
	HR (bpm)	78.0 (75.1; 80.8)	74.2 (70.3; 78.2)	80.7 (76.8; 84.6)	**0.024**
	QTc (msec)	384.0 (380.3; 387.7)	382.6 (377.4; 387.7)	385.0 (379.6; 390.4)	0.530

QTc is calculated via the Fridericia Correction Formula.

* Denotes Independent Sample T-test.

**Table 4:** QTc Comparison Within Ovulatory and Anovulatory Women during different menstrual cycle phases in the QTc Sub-study of the MOS2 during the SARS-COV-2 Pandemic.

Ovulatory Status	Variable (msec)	N	Mean ± SD	95% CI	Correlation Coefficient	T-Test *P* value	
**Ovulatory**	QTc in Mid- Follicular Phase	26	383.0 ± 12.8	377.8; 388.1	0.637	.859]*	
	QTc in Luteal Phase		382.6 ± 12.8	377.4; 387.7			
	QTc Phase Change (Luteal – Follicular)		- 0.38 ± 10.9	- 4.78; 4.02	—		.268]†
**Anovulatory**	QTc in Mid-Follicular Phase	36	381.7 ± 13.1	377.3; 386.1	0.556	.166]*	
	QTc in Pre-Menstrual phase		385.0 ± 16.1	379.6; 390.4			
	QTc Phase Change (Pre-Menstrual-Follicular)		3.3 ± 14.0	- 1.44; 8.03	—		
**Ovulatory + Anovulatory**	QTc in Mid-Follicular Phase	66	382.5 ± 12.7	379.4; 385.6	—	.212]*	
	QTc in Luteal/Pre-Menstrual phase		384.5 ± 14.5	380.9; 388.1	—		

QTc is corrected for heart rate via the Fridericia Formula.

† Denotes an Independent Samples T-test and * denotes a Paired Two-sided T-Test.

In contrast, heart rate changes were expectedly different within and between study subjects. In both groups, the heart rate (HR) was slowest and the QT interval longest during the FP. HR significantly increased by more than 5 beats per minute (bpm) from the FP to the LP/premenstrual phase in both groups (*P* < .05). Women with an anovulatory cycle also had significantly higher HRs during both the FP and the LP/premenstrual phase compared to those with ovulatory cycles (*P* < .05). The multiple linear regression analysis, QTc directionality change analysis, and sensitivity analysis were nonsignificant, and are detailed in Appendices C, D and E respectively.

### Meta-Analysis

**Fig 3:**
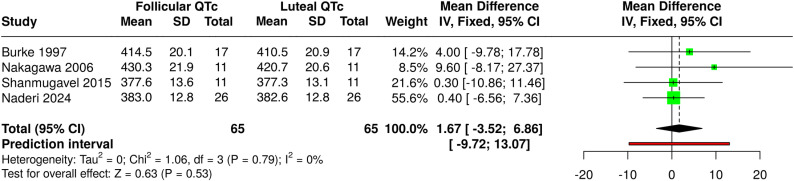
Meta-Analysis Forest Plot of QTc Difference in Ovulatory Cycles. This Random‐Effects Forest Plot Compared the Weighted Mean Difference in QTc (msec) of Follicular versus Luteal Phases in Proven Ovulatory Menstrual Cycles. The QT interval was rate-corrected using Fridericia’s Formula.

We screened seven relevant publications [6,7,10,11,30–32]; only three were eligible on the basis of ovulation documentation for being included in the meta-analysis [6,10,11]. Incorporating our original findings, we performed a pooled meta‐analysis of QTc with Fridericia’s rate correction of four studies with 65 participants (Fig 3). The meta-analysis yielded a random-effects weighted mean QTc non-significant minor shortening in the luteal versus the follicular phases, with a 1.67 msec shorter LP (95% CI: −3.52; 6.86, *P* = .53). This meta-analysis revealed no heterogeneity (I^2^ = 0%).

## Discussion

To our knowledge, this is the first prospective cohort study to compare QTc in follicular phase versus luteal phase in documented ovulatory cycles, as well as follicular phase versus the premenstrual phase in anovulatory cycles. This study also represents the largest physiological investigation of QTc and menstrual cycle phases, consisting of paired data for 62 women. It was observed that women with an ovulatory menstrual cycle experienced no meaningful QTc change from the LP to the FP. The results were confirmed through a meta-analysis of spontaneous, ovulatory cycles, showing a similar minor trend in QTc shortening during the LP. We also observed QTc prolongation from the FP to the premenstrual phase in anovulatory cycles.

The implications of these results are: 1) under normal physiological conditions, QTc changes during the menstrual cycle are trivial, and menstrual status does not need to be considered in interpreting the QT interval; 2) in a typical menstrual cycle, minor FP QTc prolongation is driven by the predominant presence and effect of estradiol, and QTc shortening during the LP is related to the effect of progesterone dominating over estradiol, although QTc changes are minimal and the interval remained stable overall; and 3) QTc prolongation during an anovulatory cycle is likely related to longer exposure to estrogen without progesterone.

Most literature on menstrual cycle-related QTc changes have relied on Bazett’s formula for rate correction or used non-validated methods for documenting ovulation; together or singly, these methods can result in inaccurate QTc assessment. Although Bazett’s formula has been the standard to rate-correct the QT interval, its clinical utility is limited due to suboptimal QT rate correction with large errors at resting and low heart rates [25–27]. Therefore, this study and the subsequent meta-analysis were the first investigation to use Fridericia’s formula [25,26]. Among the seven articles screened for our meta-analysis that purported to document ovulation, five studies used Bazett’s formula [6,7,11,30,32], with the remaining two studies acknowledging Bazett’s limitations and using other lesser-known methods for QT rate correction [10,31].

Ovulation characterization in normal-length cycles in previous QTc studies has often been inaccurate. In normal-length cycles, using sequential cycle days with day 14 considered as the day of ovulation, or backward counting from the first day of flow with days > 14 considered as the luteal phase, both fail to account for silent anovulatory cycles that may present as predictable and normal-length cycles [4,14]. It is critical, therefore, to confirm ovulation through direct, validated measures such as the urine LH surge followed by a confirmatory cycle-timed measure of progesterone (above 9.5 nmol/L or 3 ng/ml), or by validated Quantitative Basal Temperature©. To our knowledge, our research is the first to implement a more accurate rate-correction method than Bazett’s and reliably document ovulatory cycles to ensure an accurate analysis of QTc during spontaneous ovulatory and anovulatory cycles in community-dwelling women.

Despite the biological plausibility of the effects of estradiol and progesterone on cardiac repolarization, we did not detect impactful QTc changes during typical ovulatory cycles. QTc minimally decreased from FP to LP, but this change was not meaningful and remained clinically stable. The meta-analysis also revealed a similar pattern in QTc change, a finding that is consistent with the trends observed in this primary study and current literature [6,10,11]. A significant relationship may emerge when accounting for autonomic tone, as observed in two papers in which a significant QTc difference was noted only after double autonomic blockade [6,7]. Nevertheless, the directionality of QTc change remains consistent with it being shortest during the LP. These findings may be attributed to the physiology and dynamic interplay of estradiol which rises 240% above its baseline during flow, while progesterone is low for the full FP and rises to an LP peak that is 1400% above its low during flow [33]. Notably, ventricular repolarization during the LP has been shown to be driven by the change in progesterone level as opposed to estradiol [10,34]. Therefore, QTc shortening during ovulatory cycles could be attributed to the action of progesterone, as also confirmed by recent pharmacological human [8] and animal experiments [9].

There is an interplay of factors including ovarian hormone levels and autonomic tone that could influence the QT interval throughout a menstrual cycle. While studies often use exogenous hormones [8,9] and double autonomic blockade [6,7] to heighten this QTc-hormone relationship, it is imperative to study these changes under natural, physiological menstrual conditions. Our findings suggest that QTc remains relatively stable in women with an ovulatory menstrual cycle, potentially reflecting a physiological safeguard to maintain a stable cardiac rhythm. Disruption of this equilibrium, particularly through an anovulatory menstrual cycle, may lead to notable alterations in the QTc.

For the first time, it has been documented that in contrast to ovulatory cycles, women with anovulatory cycles experienced QTc prolongation from the FP to the premenstrual phases. Estradiol patterns in anovulatory cycles are unknown, but levels are about 20% lower based on a large random population study [12]. Correspondingly, there is a significant negative correlation between the serum progesterone level and the LP QTc value (r = -.41) [34]. Given this dynamic, estradiol may have an effect that lasts longer than its level at a given time, since Hulot et al. have shown that the QTc during low estradiol levels in the early FP was the same as near the mid-cycle estradiol peak [31]. This pattern of QTc prolongation during anovulatory cycles can be attributed to the dominant influence of estradiol and/or to a longer exposure interval exerting an overall greater QTc prolonging effect. These results suggest that there is a temporal dimension to the QTc- prolonging effect of estradiol, in addition to the importance of taking the estradiol-progesterone exposure ratio into account when assessing QTc change. Future research should, therefore, prioritize the exploration of such physiological disruptions that could influence the QT intervals of women and increase the risk of cardiac arrhythmias.

### Study limitations and strengths

Despite being the largest study to evaluate QTc across confirmed ovulatory menstrual cycle phases, this investigation, with its 62 participants and 26 ovulatory cycles, has a relatively modest sample size. The observed QTc changes are subtle, necessitating an exceptionally large sample size to establish a definitive statistical relationship. It has however excluded large changes in the QTc interval that would reflect dynamic arrhythmia risk.

Although the QBT© assessment of ovulation and LP lengths is a direct reflection of progesterone’s hypothalamic thermal actions which has been previously validated, we did not measure estradiol and progesterone levels. This study had markedly more ovulatory disturbances than expected because it was performed during the SARS-COV-2 pandemic [15]. Also, the ovulatory LP values in this dataset included short LP’s in which progesterone duration and quantitative exposure would be less than in normal luteal length cycles [35]. Examining women with consistent hormone states, such as those with a ≥ 10-day LP length by QBT©, could help reduce noise in the data and reveal genuine effects. We did not limit the sample to normally ovulatory cycles to avoid losing power.

The strengths of these data are: 1) the QTc timing was compared within-woman over two time points and cycle phases during a single menstrual cycle; 2) the QTc data were analyzed blinded to cycle phase; 3) ovulation was documented with a validated non-invasive QBT© method; and 4) these data were obtained in community-dwelling women.

### Clinical implications

Due to physiological differences in estradiol exposure, women experience longer QTc values compared to men, and any QT prolongation, either hormonal or drug-induced, can increase the risk of ventricular arrhythmias [36]. The results of this investigation suggest that documented ovulation with normal and short luteal phases has relatively little or no effect when examining QTc in premenopausal women.

## Conclusion

In this prospective study, the QTc changes in women with an ovulatory menstrual cycle showed minimal variation, with the meta-analysis also reflecting the same pattern in minimal luteal phase QTc shortening. The sequential effects of estradiol and progesterone, which respectively prolonged and shortened the QTc, were very minor, non-significant changes during the entire cycle. QTc change during anovulatory cycles were also trivial, with minor non-significant QTc prolongation from mid-FP to the premenstrual phase, owing to longer estradiol exposure without the presence of progesterone.

## Supporting information

S1 Appendix Menstrual Cycle Diary©. (DOCX)

S2 Appendix Participant Pre-ECG Questionnaire. (DOCX)

S3 Appendix Multiple Regression Analysis. (DOCX)

S4 Appendix QTc Directionality Change Analysis. (DOCX)

S5 Appendix Sensitivity Analysis. (DOCX)
