# Supplementary material for: Ovulatory and anovulatory cycle phase influences on QT interval dynamics during the menstrual cycle
Source: PLoS One. 2025 May 16;20(5):e0320846. doi: 10.1371/journal.pone.0320846 (PMC12083801; doi:10.1371/journal.pone.0320846)
Supplement: S1 Appendix — (DOCX) [file pone.0320846.s001.docx]

**S1 Appendix**

Menstrual Cycle Diary^©^

Participants completed the Menstrual Cycle Diary^©^ daily; an 18-item instrument scored 0-4 for experiences such as flow and cramp intensity, and on a 5-part letter-indicated scale centered around “U” for usual for items such as feeling of self-worth or outside stress. Although this study did not utilize the Diary's variables, it provided a structured method to document cycle lengths, and for participants to record their daily first morning temperatures. The form is accessible at: <http://www.cemcor.ca/resources/daily-menstrual-cycle-diary>.
