# Supplementary material for: Ovulatory and anovulatory cycle phase influences on QT interval dynamics during the menstrual cycle
Source: PLoS One. 2025 May 16;20(5):e0320846. doi: 10.1371/journal.pone.0320846 (PMC12083801; doi:10.1371/journal.pone.0320846)
Supplement: S2 Appendix — (DOCX) [file pone.0320846.s002.docx]

**S2 Appendix**

Participant Pre-ECG Questionnaire

The Participant Pre-ECG Questionnaire was administered to each study participant. A sample is provided below.

There are various factors that can influence the results of an electrocardiogram, also known as an “ECG.” This questionnaire is designed to help researchers understand why there may be variation between your ECG results. Your responses will be kept confidential.

1. Do you smoke/vape cigarettes?

☐ NO

☐ YES

- If yes, when was the last time you smoked/vaped? ___________

2. Are you currently taking any medications?

☐ NO

☐ YES

- If yes, please specify: ___________

3. Do you do strenuous exercise?

☐ NO

☐ YES

- If yes, when was the last time you did very hard exercise? ___________

4. Considering your whole life in the past week, how stressed are you at the current moment? (Scale 1-10)

(Not Stressed) 1 2 3 4 5 6 7 8 9 10 (Very stressed)

5. Have you had coffee or a (high caffeine) “energy drink” today?

☐ NO

☐ YES

- If yes, how many cups? ___________
- b. How long ago was your last drink? ___________
