# Supplementary material for: Ovulatory and anovulatory cycle phase influences on QT interval dynamics during the menstrual cycle
Source: PLoS One. 2025 May 16;20(5):e0320846. doi: 10.1371/journal.pone.0320846 (PMC12083801; doi:10.1371/journal.pone.0320846)
Supplement: S3 Appendix — (DOCX) [file pone.0320846.s003.docx]

**S3 Appendix**

Multiple Regression Analysis

To assess the role of other variables that could influence the QTc difference between the follicular and luteal phases, a multiple linear regression analysis was performed using R version 4.3.0. Adjustments for waist circumference and cycle phase heart rate variability in the multivariate regression analysis resulted in a mean QTc phase difference of 2.4 msec (95% CI -4.8; 10.2) between the ovulatory and anovulatory cycles. This relationship was nonsignificant (*P* =.472) and accounted for only 9.3% of the variance.
