# Supplementary material for: Ovulatory and anovulatory cycle phase influences on QT interval dynamics during the menstrual cycle
Source: PLoS One. 2025 May 16;20(5):e0320846. doi: 10.1371/journal.pone.0320846 (PMC12083801; doi:10.1371/journal.pone.0320846)
Supplement: S4 Appendix — (DOCX) [file pone.0320846.s004.docx]

**S4 Appendix**

QTc Directionality Change Analysis

We explored the directionality change of QTc values across the follicular-luteal phase by categorizing QTc changes using the Food and Drug Administration (FDA) accepted cut-off of 5 msec as a meaningful difference. [1] In this discrete analysis (eTable 1), QTc changes were divided into four categories: an increase of more than 5 msec, a decrease of more than 5 msec, an increase between 0 and 5 msec, and a decrease between 0 and 5 msec. To showcase the changes, a Delta Plot was created using Microsoft Excel.

The directionality of QTc changes based on the FDA accepted 5 msec cut-off remained unchanged between ovulatory and anovulatory cycles. However, anovulatory cycles tended to exhibit greater QTc prolongation (>5 msec) and lesser QTc premenstrual shortening (<5 msec).

**S4 Table. Categorized QTc Change across the Follicular-Luteal Phases in Ovulatory Cycles: Figures A and C Illustrate the Directionality of QTc Changes in Anovulatory Cycles and Figures B and D in Ovulatory Cycles.** P value was calculated via a paired T-Test.

**
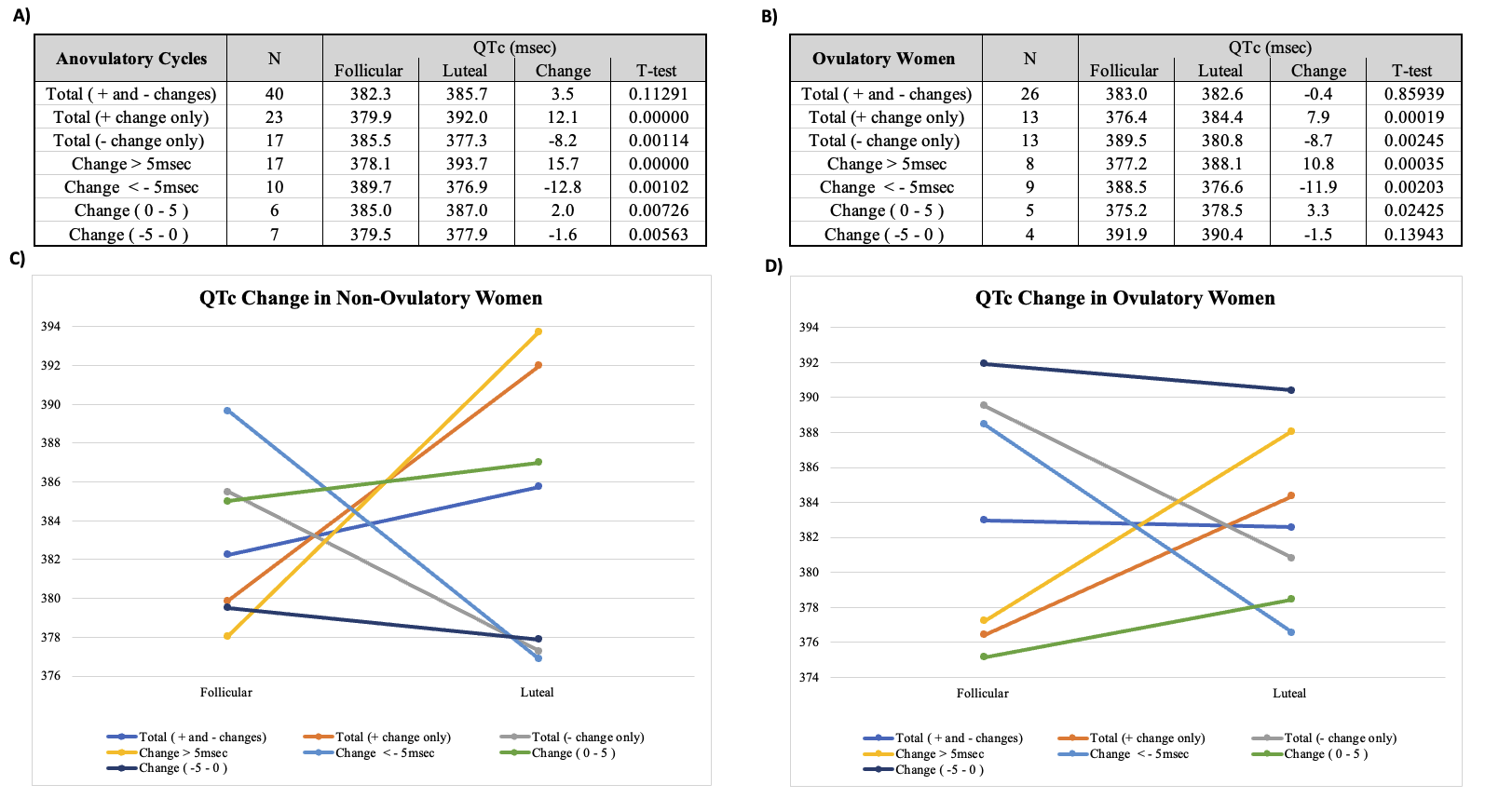
**

**References for Appendix S4**

1. Food and Drug Administration, HHS. International Conference on Harmonisation; guidance on E14 Clinical Evaluation of QT/QTc Interval Prolongation and Proarrhythmic Potential for Non-Antiarrhythmic Drugs; availability. Notice. *Fed Regist*. 2005;70(202):61134-61135.
