# Supplementary material for: Ovulatory and anovulatory cycle phase influences on QT interval dynamics during the menstrual cycle
Source: PLoS One. 2025 May 16;20(5):e0320846. doi: 10.1371/journal.pone.0320846 (PMC12083801; doi:10.1371/journal.pone.0320846)
Supplement: S5 Appendix — (DOCX) [file pone.0320846.s005.docx]

**S5 Appendix**

Sensitivity Analysis

Two sensitivity assessments were performed (eTable2). The first analysis involved excluding women with a Quantitative Basal Temperature^©^ (QBT) cycle phase mean difference of only 0.1 °C (or within the digital thermometer error). This assessment included only women with a difference of 0.2 or 0.3 °C, suggesting a high progesterone effect. Only one woman was excluded, having a mean follicular-to-luteal temperature increase of 0.1 ºC. The exclusion resulted in a similar, nonsignificant luteal phase QTc change (*P* = .753).

The second analysis assessed QTc measurements that were recorded at least three days after the QBT© temperature shift, to account for readings that were too early in the luteal phase for progesterone to have an effect. The familywise Type 1 error rate was 0.05. Statistical software SPSS version 29 (Armonk, NY: IBM Corp) was used for these analyses. Six women in the ovulatory group with a QTc measurement within three days of the QBT© temperature shift were excluded. The sequential removals resulted in a nonsignificant difference in the luteal phase QTc (*P* = .965).

**S5 Table. Sensitivity Analysis of Thermometer Variability and QTc Changes.** Sensitivity analysis for variability within the digital thermometers compared to a mercury control instrument and change in QTc when measured within 3 days of the Quantitative Basal Temperature^©^ (QBT) shift in data from MOS2 during the SARS-COV-2 Pandemic.

| **Sensitivity Analysis** | **QTc (milliseconds)** | **Mean ± SD** | ***P* Value ***** |
| --- | --- | --- | --- |
| Removal of one ovulatory woman with a temperature difference of 0.1 ºC on the digital thermometer (n = 25) | Mid follicular phase | 382.8 ± 13.0 | .753 |
|  | Luteal phase | 382.1 ± 12.7 |  |
| Removal of six ovulatory women with a QTc measurement within 3 days of the QBT shift (n = 20) | Mid follicular phase | 384.5 ± 12.9 | .965 |
|  | Luteal phase | 384.6 ± 12.7 |  |

***Paired T-test
